# The Effect of Perioperative Fluid Management and Operative Modifications on Mortality and Morbidity in Patients Undergoing Pulmonary Endarterectomy

**DOI:** 10.21470/1678-9741-2021-0009

**Published:** 2023

**Authors:** Akın Arslan, Mehmed Yanartaş, Serpil Taş, Nilgün Bozbuğa, Bedrettin Yıldızeli

**Affiliations:** 1 Department of Cardiovascular Surgery, Sakarya Research and Training Hospital, Sakarya, Turkey.; 2 Department of Cardiovascular Surgery, Başakşehir Çam and Sakura City Hospital, İstanbul, Turkey.; 3 Department of Cardiovascular Surgery, Koşuyolu Training and Research Hospital, İstanbul, Turkey.; 4 Department of Cardiovascular Surgery, Istanbul Faculty of Medicine, Istanbul University, İstanbul, Turkey.; 5 Department of Thoracic Surgery, Marmara University Pendik Training and Research Hospital, İstanbul, Turkey.

**Keywords:** Pulmonary Embolism, Endarterectomy, Thrombosis, Cardiopulmonary Bypass, Intensive Care Units, Morbity

## Abstract

**Introduction:**

Chronic thromboembolic pulmonary hypertension (CTEPH) is a severe disease treated with pulmonary endarterectomy. Our study aims to reveal the differences in liquid modalities and operation modifications, which can affect the patients’ mortality and morbidity.

**Methods:**

One hundred twenty-five patients who were diagnosed with CTEPH and underwent pulmonary thromboendarterectomy (PTE) at our center between February 2011 and September 2013 were included in this retrospective study with prospective observation. They were in New York Heart Association functional class II, III, or IV, and mean pulmonary artery pressure was > 40 mmHg. There were two groups, the crystalloid (Group 1) and colloid (Group 2) liquid groups, depending on the treatment fluids. P-value < 0.05 was considered statistically significant.

**Results:**

Although the two different fluid types did not show a significant difference in mortality between groups, fluid balance sheets significantly affected the intragroup mortality rate. Negative fluid balance significantly decreased mortality in Group 1 (P<0.01). There was no difference in mortality in positive or negative fluid balance in Group 2 (P>0.05). Mean duration of stay in the intensive care unit (ICU) for Group 1 was 6.2 days and for Group 2 was 5.4 days (P>0.05). Readmission rate to the ICU for respiratory or non-respiratory reasons was 8.3% (n=4) in Group 1 and 11.7% (n=9) in Group 2 (P>0.05).

**Conclusion:**

Changes in fluid management have an etiological significance on possible complications in patient follow-up. We believe that as new approaches are reported, the number of comorbid events will decrease.

## INTRODUCTION

Chronic thromboembolic pulmonary hypertension (CTEPH) is a severe disease with high mortality thought to be the result of recurrent pulmonary embolism (PE) episodes from a venous focus or thrombosis originating from the native structure of the pulmonary artery. Progressive right heart failure is observed in almost all patients and it is fatal^[1]^. Because of the disease’s insidious onset, the diagnosis is usually caused by shortness of breath and right heart failure signs. When pulmonary artery pressure is > 40 mmHg, it means that the late phase has been reached. When the mean pulmonary artery pressure is > 50 mmHg in thromboembolic disease, the three-year mortality rate reaches 90%^[1]^. The estimated incidence of CTEPH after acute pulmonary embolism (APE) is 0.5-3.8%^[2]^. Deep vein thrombosis (DVT) has been observed in 90% of clinically defined PE cases, but DVT is clinically asymptomatic in two-thirds of patients with DVT and CTEPH^[3,4]^. Most cases diagnosed as DVT and APE are treated with medical treatment. According to the guidelines published in 2011 in APE cases, there is no Class 1 surgery indication unless the main pulmonary artery is massively occluded, causes low flow, and there is life-threatening right heart failure^[5]^.

Conversely, the main element of the treatment of CTEPH is surgical removal of the thromboembolic material with the distal ends, along with an endarterectomy technique. In this form, CTEPH is a surgically treatable type of pulmonary hypertension. In pediatric patients, CTEPH and successful pulmonary thromboendarterectomy (PTE) series are available too^[6]^. The thrombosis associated with the CTEPH is the presence of lupus anticoagulant, increased antiphospholipid antibodies, and increased factor VIII^[7]^. Behçet’s disease has been reported as another possible risk factor^[8]^. Deficiencies of protein C, protein S, and antithrombin III or factor V Leiden and factor II mutations are no longer shown among high-risk factors in the development of CTEPH^[9]^.

The definitive treatment of CTEPH patients is PTE. Surgical mortality has been reported between 4.1% and 10.3% in recent years^[10]^. Our aim in this study is to reveal the effects of the operative modifications we performed and perioperative fluid management on increasing surgical success levels.

## METHODS

Study data were obtained by retrospective examination and prospective observation methods. Patients’ preoperative characteristics are shown in Table 1. We were based on the Jamieson Surgical Thromboembolic Classification criteria (Table 2). This classification includes type 1, type 2, type 3, and type 4. Type 4 patients were considered inoperable and excluded from the study. One hundred twenty-five patients who were diagnosed with CTEPH in our hospital in 2011 and 2013 and underwent PTE operation were included in the study. Patients were divided into two groups according to the use of crystalloid (Group 1) and colloid (group 2) fluid in the perioperative period.

**Table 1:** Patients’ preoperative characteristics.

Characteristics	Group 1 (n = 48)	Group 2 (n = 77)	*P*-value^1^
Age (years)	45.6±13.1	50±13.8	0.087
Gender (female/male)	28/20	47/30	
Concomitant factors			
DVT history	22 (45.8%)	30 (39%)	
Hypertension	21 (43.8%)	29 (37.7%)	
Diabetes mellitus	10 (20.8%)	10 (13%)	
Smoking	18 (38.5%)	19 (24.7%)	
Pulmonary artery pressure (mm Hg)			
Systolic	75±26.1	74±27.3	0.918
Diastolic	31.5±13.2	32.5±14.2	0.668
Mean	46±16.6	46.5±17.2	0.857
Pulmonary vascular resistance (dynes/sec/cm^5^)	749.4±363.7	876.5±477	0.117
Cardiac output (L/min)	3.91±0.94	4.14±1.38	0.319
Tricuspid insufficiency	2.4±1.2	2.3±1.1	0.472
NYHA classification			0.439
II	11 (22.9%)	24 (31.2%)	
III	29 (60.4%)	45 (58.4%)	
IV	8 (16.7%)	8 (10.4%)	

Number and standard deviation ratios of the data are shown as (±)

1 Statistically significant *P*-value < 0.05

DVT=deep vein thrombosis; NYHA=New York Heart Association

**Table 2:** Jamieson Surgical Thromboembolic Classification.

Characteristics	Types	Group 1 (n = 48)	Group 2 (n = 77)	*P*-value^*^
Jamieson; both sides for different lesions				0.536
	No thromboembolic disease (*e.g.*, tumour)	1 (2.1%)	1 (1.4%)	
	Type I	22 (45.8%)	31 (41.9%)	
	Type II	18 (37.5%)	36 (48.6%)	
	Type III	6 (12.5%)	6 (8.1%)	
	Type IV	1 (2.1%)	0 (0%)	
Jamieson; both sides for a similar lesion				
	Bilateral Type I	13 (27%)	7 (9%)	
	Bilateral Type II	9 (18.7%)	29 (37.6%)	
	Bilateral Type III	3 (6.2%)	5 (6.5%)	
	Bilateral Type IV	1 (2%)	0 (0%)	

* Statistically significant *P*-value < 0.05

Group 1 patients were given crystalloid solutions as fluid replacement. Standard aortic cross-clamp application and standard cooling and rewarming techniques were used as a surgical modality. At the beginning of cardiopulmonary bypass (CPB), 1500 ml of Ringer solution (Neofleks®) as prime crystalloid solution and additional standard solution were used at a dose of 20% mannitol 2.5 ml/kg; 0.09% isotonic solution (Neofleks®) or Ringer solution (Neofleks®) was preferred during postoperative treatment.

In Group 2 patients, colloid solutions were preferred as fluid replacement. During the surgical procedure, prolonged cooling and rewarming (≈ 90 minutes) were applied with the short-term and intermittent aortic cross-clamp application. In prime colloid solution, Voluven® 6% (Hydroxyethyl Starch, Hepp 130/0 in isotonic sodium chloride solution-Fresenius Kabi®) 1500 ml and standard additional solution 20% mannitol 2.5 ml/kg were used.

### Pulmonary Endarterectomy

Since this pathology is bilateral in most of the patients, an endarterectomy operation should also be performed bilaterally. Median sternotomy is the most reasonable way for a surgical intervention involving both pulmonary arteries. In unilateral approaches to thoracotomy, an excellent surgical opinion may not be achieved even if the opposite pulmonary artery is clamped. Collateral circulation is provided not only with bronchial arteries but also with diaphragmatic, intercostal, and pleural vessels. Therefore, the surgical field in thoracotomy can be quite bloody. An excellent and bloodless field of view is necessary to identify an adequate endarterectomy plane and then follow the endarterectomy material up to the subsegmental vessels. So, total circulatory arrest is a beneficial method. Although operations rarely performed without total circulatory arrest (TCA) are reported, the bloodless environment is created by entering the TCA due to the current bronchial collateral flow. The PEACOG study on this issue said that the TCA method was safer than cerebral perfusion^[11]^.

### Surgical Technique

After standard median sternotomy, the pericardium was opened, and the heart was reached. The aorta and both vena cava were cannulated for total CPB. Cardiac decompression was achieved through CPB after cannulation. A line was placed 1 cm below the pulmonary valve for drainage of the pulmonary artery (the point also marks the arteriotomy). When CPB was initiated, cooling of the head and body was started from the outside. The blood was cooled with the heat exchanger pump. Cooling process lasted 45 minutes-1 hour as planned. An additional cannula was placed in the left atrium through the right upper pulmonary vein when ventricular fibrillation occurred. During the cooling period, the aorta and the right pulmonary artery can be fully mobilized with some preparatory dissections. When the patient reaches a temperature of 20 °C, it is placed a cross-clamp, and the cold cardioplegia solution is administered in a single dose (1 L). Aortic cross-clamp application and cooling and rewarming period were performed with different modifications in both groups. In the first group, continuous cross-clamp application and standard cooling and rewarming period was applied. Group 2 used the intermittent cross-clamp application and a prolonged cooling and rewarming period. The right pulmonary artery incision should be initiated by the medial side of the superior vena cava. A microtome knife (Jamieson dissector) is used in the posterior dissecting line.

**Fig. 1:**
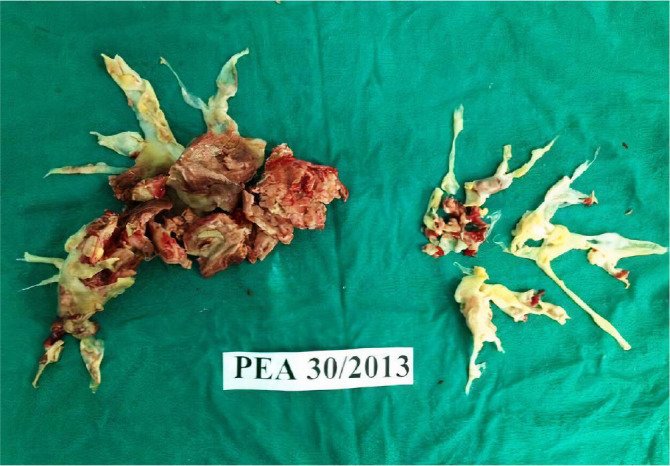
Operation material extracted from a patient. PEA=pulmonary endarterectomy

The ideal layer can be easily peeled and seen as a pearl white. There should be no traces of yellow plaque. If the dissecting flap is deep, the reddish or pink appearance will indicate adventitia. When a full layer is obtained in the correct plan, endarterectomy is performed by the eversion technique. The whole specimen is “tail-shaped” and must be freely reduced (Figure 1). The left-sided endarterectomy process has almost similar characteristics to the right side in terms of applicability. Total circulatory arrest time is identical too. After the surgical procedures were completed, the rewarming time was planned to be 90-120 minutes. The temperature did not exceed 37 °C.

### Statistical Analysis

IBM Corp. Released 2012, IBM SPSS Statistics for Windows, Version 21.0, Armonk, NY: IBM Corp. program was used for statistical analysis. Numerical data were expressed as mean ± standard deviation and categorical data as a percentage (%). In comparing numerical values between the two groups, the Student’s *t*-test and Pearson’s chi-squared test were used to compare categorical variables. Multivariate stepwise linear regression analyses were used to determine independent predictors of increased cardiac output. *P*-value < 0.05 was considered statistically significant.

## RESULTS

The mean age of Group 1 patients was 45.6 years. The female/male ratio was 28/20. Of the patient population, 20.8% were diabetic, 43.8% were hypertensive, and 45.8% had a known history of DVT. The smoking rate was 38.5%. As an additional procedure, mitral valve repair (2.08%) was performed in one patient, and tricuspid de Vega annuloplasty (6.25%) and atrial septal defect/patent foramen ovale closure (12.5%) were performed in three patients. Coronary artery bypass grafting (CABG) (4.16%) was performed in two patients. Right atrial mass excision (4.16%) was performed in two patients. The mean age of Group 2 patients was 50 years. The female/male ratio was 47/30. Diabetes mellitus in 13%, hypertension in 37.7%, and DVT history known in 39% of the patients were present. Smoking rate was 24.7%. In addition to PTE, CABG was performed in three patients (3.89%), and sternal bar removal was performed in one patient (1.29%).

**Fig. 2:**
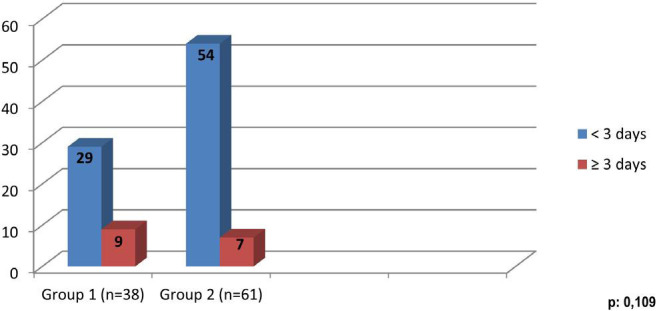
Ventilatory support times.

Statistically, there was no difference between the two groups in terms of demographic data. In general, there was no statistically significant difference between the two groups compared to the mortality rates of their groups (*P*>0.05). When the effect of fluid therapy on the ventilatory-assisted period was examined, there was no statistically significant difference between the two groups (*P*>0.05) (Figure 2). The length of stay in the intensive care unit (ICU) of the patients in both groups was examined. The mean duration of stay in the ICU for Group 1 patients was 6.2 days, and the mean duration for Group 2 patients was 5.4 days. There was no statistically significant difference between the groups (*P*>0.05). After discharge from the ICU, the readmission rate to the ICU for respiratory or non-respiratory reasons was 8.3% (n=4) in Group 1 patients and 11.7% (n=9) in Group 2 patients. These rates are not statistically significant (*P*>0.05). The total length of hospital stay in Group 1 is 11.6 days and in Group 2 is 16.1 days. This difference was statistically significant (*P*<0.05).

When the data in Table 3 were examined, the fluid balance of Group 2 patients in whom colloid fluid was preferred was statistically significantly lower. The overall fluid balance after CPB was a positive balance in both groups. The critical point here is that crystalloid solutions can be easily extracted by extravasation and increase the overall body fluid balance. In other parameters, partial pressure of oxygen (PaO₂) and oxygen saturation (SaO₂) values of Group 2 patients were statistically significantly higher than Group 1 patients. When other parameter was examined, the osmolarity values of the fluids used in both groups were almost equal (308,8/308 mOsm/L). It is thought that Voluven® in Group 2 resulted in relatively low osmolarity due to its ability to provide volume in the intravascular area (4-6-hour plateau style). Although the generally preferred liquid type does not differ statistically significantly between the two groups’ mortality rates, it is interesting that the positive or negative fluid balance sheet differs between groups in terms of mortality. Namely, in Group 1 patients, the negative fluid balance sheet was determined to reduce mortality statistically significantly (*P*<0.01). Nevertheless, the positive or negative fluid balance sheet in Group 2 patients had no statistically significant mortality (*P*>0.05).

**Table 3:** Comparison of perioperative fluid treatment and ventilator values according to groups.

Characteristics	Group 1 (n = 48)	Group 2 (n = 77)	*P*-value^1^
General fluid balance after CPB (ml)	1058±760	705±760	0.017
PaO₂ value after CPB	335±152	267±100	0.005
SaO₂ value after CPB	98.5±2.9	99.6±1.1	0.007
Osmolarity after CPB	292.3±10.5	286.6±8.7	0.013

1 Statistically significant *P*-value < 0.05

CPB=cardiopulmonary bypass; PaO₂=partial pressure of oxygen; SaO₂=oxygen saturation

### Hemodynamic Parameters

After CPB, in groups, inotrope and intra-aortic balloon pump (IABP) support requirements were statistically different. This difference has been in favor of Group 2. Cardiac output, which is the most critical cardiac performance indicator, was significantly increased in Group 2 patients. Also, the need for inotrope and left ventricular assist device was decreased. These results were evaluated with multivariate analysis of variance. Besides, the dramatic decline of the pulmonary vascular resistance (PVR) was also observed in the two groups. In particular, Group 2 results were statistically significantly lower than Group 1 (Table 4). PVR is a critical predictor for mortality, as Madani et al. have stated in their study^[10]^.

**Table 4:** Preoperative/postoperative hemodynamic results and operating room times.

Characteristics	Group 1 (n = 48)	Group 2 (n = 77)	*P*-value^1^
PVR (dynes/sec/cm^5^)			
Preoperative	766.5±368.8	884.8±488.6	0.165
Postoperative	540.4±306.1	370.3±138.4	< 0.001^a^
Cardiac output (L/min)			
Preoperative	3.93±0.98	4.10±1.47	0.303
Postoperative	4.80±1.81	6.33±1.69	< 0.001^a^
Mean pulmonary artery pressure (mmHg)^2^			
Preoperative	73.6±26.5	70.7±28.2	0.568
Postoperative	40.1±12.9	37.6±11.9	0.271
Tricuspid insufficiency^3^			
Preoperative	2.36±1.22	2.30±1.06	0.789
Postoperative	1.40±0.93	1.05±0.72	0.024
Operation data			
CPB time (min)	170.8±47.6	189.9±31.1	0.008
Cross-clamping time (min)	106.8±29.9	17.2±23.7	< 0.001^a^
TCA time (min)			
Right pulmonary TCA	10.4±5.2	14.4±5.8	< 0.001^a^
Left pulmonary TCA	10.1±5.4	13±5.2	0.005
Total TCA	20.1±8.4	25,6±10.8	0.003

a Very high statistical significance

1 Statistically significant *P*-value < 0.05

2 The results are based on the echocardiographic evaluation

3 Patients who performed the tricuspid de Vega annuloplasty were not included

CPB=cardiopulmonary bypass; PVR=pulmonary vascular resistance; TCA=total circulatory arrest

## DISCUSSION

Although CTEPH is a disease that involves complicated processes as diagnosis and treatment, mortality rates are decreasing thanks to the updated pharmacological and surgical treatment options, and the projected lifetimes are gradually increasing^[11,12]^.

In this study, we compared several perioperative applications previously described and quoted in the guidelines. In our study, we examined the results of some modifications in perioperative applications. First, the results obtained using crystalloid and colloid in selecting CPB prime solutions and postoperative treatment fluid were examined. First of all, the reasons for an extended stay in ICU and returning to the ICU were different. The importance of knowing this difference, namely the etiology, saves time in solving the problem. Another consideration was the comparison between the two groups’ aortic cross-clamping (ACC) times and cooling and rewarming periods. Standard cooling, continuous cross-clamp application, and standard rewarming period were applied in Group 1 patients. In Group 2 patients, prolonged cooling and rewarming (90 minutes for each) and intermittent short-term cross-clamp applications were performed. When CPB was terminated, inotrope and IABP support requirements were different between the groups. This difference is in favor of the Group 2. In addition to factors affecting particularly morbidity, such as the preferred fluid type and postoperative fluid balance, factors such as the aortic cross-clamp application method and the total operation time significantly affect surgical success. It is thought that the surgical technique or fluid management used affects morbidity rather than mortality. In their comprehensive case series, Madani et al. have publicly emphasized that preoperative and postoperative PVR values are necessary to predict mortality^[10]^. Besides, the mean ACC times published by this team were observed to be longer than our ACC times (especially Group 2). However, the mortality data that Madani et al. reported seemed lower^[10]^. These differences in results support that operation time alone is not a sufficient predictor for mortality.

### Limitations

Due to the retrospective part, which is one of the two components that made up the design of our study, the expected limitations of retrospective studies were also valid here. In addition, although we had data such as PVR measurements, there were no specific tests with which we could objectively compare these patients during the hospitalization period. At the same time, the information on whether the patients received medical treatment until the operation period may not always be objective.

## CONCLUSION

This study shows that although crystalloid and colloid fluid regimens do not significantly differ in inpatient mortality, colloid fluid preference positively affects morbidity. Furthermore, the shortening of the ACC time improves cardiac performance and decreases the mortality rate. In this study, the value of PVR predicting mortality was statistically lower in the group who used colloid fluid, resulting in a multifactor advantage package. In other words, the results of the patients named Group 2 have lower PVR, higher PaO2 and SaO2, higher cardiac output, shorter duration of ventilator dependency, and less inotropic support.
